# Morphological differences between aerial and submerged sporidia of bio-fongicide *Pseudozyma flocculosa* CBS 16788

**DOI:** 10.1371/journal.pone.0201677

**Published:** 2018-08-01

**Authors:** Omran Zaki, Frederic Weekers, Philippe Compere, Philippe Jacques, Philippe Thonart, Ahmed Sabri

**Affiliations:** 1 Microbial Processes and Interactions (MiPI), TERRA Teaching and Research Centre, Gembloux Agro-Bio Tech, University of Liège, Gembloux, Belgium; 2 Artechno sa, Isnes, Belgium; 3 Biologie Générale et Morphologie Ultrastructurale, University of Liège, Liège, Belgium; US Department of Agriculture, UNITED STATES

## Abstract

*Pseudozyma flocculosa* is a fungus very useful and highly efficient as a biocontrol agent against powdery mildew. The reproduction of this fungus occurs exclusively by asexual production of conidia or sporidia that are the most suitable form for agricultural use and seems to be the most resistant to storage conditions. Despite the advantages offered by *P*. *flocculosa* in biological control, the use of this fungus use remains largely limited compared to that of chemical fungicides, at least partly due to the difficulty to obtain sporidia resistant to adverse environmental stresses in submerged culture conditions. Under solid-state and submerged-state cultivation, *P*. *flocculosa* strain CBS 16788 produced different types of sporidia. The submerged sporidia (SS) appeared relatively uniform in size, which was 15,4 ± 1,6 μm μm long, and 2,8 ± 0.8 μm wide. The aerial sporidia (AS) varied in shape and size, with a mean length of 8,2 ± 3 μm and width of 2,3 ± 0.6 μm. Under scanning and transmission electron microscopy, the cell wall of submerged sporidia was thinner than that of aerial spores, and the surface was smooth in contrast to the aerial sporidia that had a tendency to have verrucous, brittle surface characteristics. The thickness of the aerial sporidia wall is due to the presence of an outer layer rich in melanin. The sporidia germination was compared on YMPD (yeast extract, malt extract, soy peptone, dextrose and agar) coated coverslips. The aerial sporidia did not show germ tubes until 5 h of incubation, while the submerged sporidia showed many germ tubes after the same time. The resistance against the adverse environmental conditions in relation to the type of sporidia of *P*. *flocculosa* is discussed.

## Introduction

*Pseudozyma flocculosa* is an epiphytic fungus isolated from clover leaves infected with powdery mildew (*Erysiphe polygoni*) [1]. It belongs to the *Basidiomycota* class and to the order of Ustilaginales [2,3].

Accordingly, the use of *P*. *flocculosa* has received a great attention as a biological control agent against powdery mildew [4,5]. Powdery mildew diseases affect many plants species and is predominant in important greenhouse crops: roses attacked by *Sphaerotheca pannosa*, cucumbers by *Sphaerotheca fulginea*, and tomatoes by *Oidium neolycopersici* [6–8].

Regarding the mode of action of *P*. *flocculosa*, recently, a transcriptomic analysis of the tripartite interaction *P*. *flocculosa*–*Blumeria graminis*–*Hordeum vulgare* has highlighted a complex phenomenon involving the three partners and qualified as hyperbiotrophy [9].

The production of *P*. *flocculosa* relies on the formation of asexual spores. The asexual spores are called conidia in *Deuteromycota* (*e*.*g*. *Fusarium sp* and *Aspergillus niger*) and sporidia in *Basidiomycota* (*e*.*g*. *P*. *flocculosa*, *Ustilago maydis*) [9–11]. Conidia and sporidia are the principal means of protection and conservation of the fungal genome, thanks to their resistance to hostile environmental conditions such as extreme temperature, ultraviolet radiations or low relative humidity. For example, *A*. *niger* conidia maintain their germination ability after exposition to temperature of 98°C, or after autoclaving for one hour at 120°C [12,13].

The resistance of conidia or sporidia arises from their particularities, *i*.*e*. the compartmentalization of cytoplasm and a thick wall rich in melanin molecules on its outer part [14,15]. The compartmentalization enables the conidia or sporidia to store the compounds necessary for their germination after their dormancy state. Melanin plays a very important role in the protection of fungal cells and other structures against unfavorable environmental factors such as UV rays, lytic enzymes and toxic metals [16,17]. Melanin is involved in pathogenesis by increasing the resistance of pathogenic fungi [18–22].

In order to produce the sporidia of *P*. *flocculsa*, two techniques are commonly used: (1) solid-state fermentation (SSF) using a solid substrate, *e*.*g*. wheat bran, rice on an inert support such as polypropylene foam. This technique is similar to the natural habitat of a fungi life cycle, and (2) liquid-state fermentation (LSF). The latter is preferred for the production of spores on a large scale due to its low costs, relatively short time of culture, equipment availability, and the number of studies that have focused on improving this type of production.

Despite all the advantages of LSF, the conidia or sporidia produced by this technique largely differ from those produced by SSF. For example, the conidia of *Trichoderma harzianum* and *Verticillium lecanii* from LSF show a weak resistance against UV radiation and low longevity compared to conidia from SSF [23–26].

Several studies have named “blastospores”, the non-resistant spores harvested from submerged cultures [27,28]. In our study, this term is not appropriate because “blastospore” refers to on the type of sporogenesis and not to the culture method (LSF or SSF) [29]. This is why we preferred to use the term “aerial sporidia (AS)” for sporidia of *P*. *flocculosa* obtained from SSF and “submerged sporidia (SS)” for those obtained from LSF [25].

To better understand the reasons of the weakness of the SS of *P*. *flocculosa*, the present study focuses on morphological and anatomical comparison between SS and AS using several microscopic approaches. Furthermore, the knowledge of the type of sporogenesis cycle in *P*. *flocculosa* could help us to understand the phenomenon of sporogenesis.

## Materials and methods

### Microorganism

*Pseudozyma flocculosa* (CBS 167.88), isolated in 1986 from *Trifolium pratense* leaves infected with *Erysiphe poligoni* by Traquair *et al*. [1], was used throughout this study.

The culture was maintained on YMPD solid medium (yeast extract 0.3% w/v, malt extract 0.3´% w/v, soy peptone 0.05% w/v, dextrose 1% w/v and agar 2% w/v) at 4°C [30]. Sporidia were harvested from cultures grown for 8 days at 28°C by flooding the agar surface with sterile saline solution containing a 0,02% w/v of Tween 80. For long term storage, the sporidia were kept at -80°C using a bead cryogenic vials (Sigma-Aldrich, Germany). For immediate inoculation, aliquots of 1 ml suspension were stored at 4°C in tubes (about 10^6^ spores/ml).

In liquid-state fermentations, 100 ml YMPD (without agar) was inoculated with 1 ml of sporidia suspension, and was cultivated for 4 days at 28°C, 150 rev/min.

In the solid-state fermentation, 200 g of substrate (100 g wheat bran in 100 ml water) was sterilized and inoculated with 10 ml of culture suspension, and cultivated for 12 days at 28°C.

### White light and fluorescence microscopy

Ten μl of AS from a one-week-old culture suspended in sterile saline solution with 0,02% w/v of Tween 80 and 10 μl of SS from 4-day-old culture was dropped directly onto a glass slide and covered with a coverslip.

Nile red fluorescent dye (1 mg/ml solution in acetone; Sigma-Aldrich, Germany) was used to detect the lipid reserves in the cytoplasm of sporidia. The dye was first dissolved in 1 ml of acetone, then added at the ratio 1/10 (v/v) to the sporidia suspension and incubated in the dark at room temperature for one hour. The sporidia were harvested by centrifugation at 4000 rpm, twice washed with distilled water and re-suspended in a sodium acetate solution buffer (5mM) [31]. The sporidia samples were observed in white light and red fluorescence microscopy (microscope Zeiss-Axioskop 2 MOT). The microscope was fitted with a camera-type Axiocam HRC color Carl Zeiss technology, operating with the AxioVision 3.1. software. Size and length measurements were performed on 50 cells of each of AS and SS.

### Scanning electron microscopy (SEM)

Submerged sporidia harvested from 4-day-old liquid culture and aerial spores from a one-week-old culture on YMPD gel were fixed for one hour in 2.5% of glutaraldehyde 0.1 M phosphate buffered saline (PBS) at pH 7.4. After fixation, the suspensions were centrifuged and washed thrice for 5 min in distilled water to remove chemicals. Sporidia were then freeze-dried and mounted on aluminum stubs using double-sided carbon tape and Pt-coated in a Balzers SCD 030 sputtering unit. For AS and sporogenesis cycle studies, samples were taken from Petri dish, left to air-dry on the stubs without fixation, and Pt-coating.

Scanning electron micrographs were taken at graded magnifications (between x1000 and x10000) in a JEOL JSM 840-A Scanning Electron Microscope working at 20 kV accelerating voltage and with the Orion 6.60.6. software (E.L.I. Vision) for digital image capture.

### Transmission electron microscopy (TEM)

Two samples were used for this comparative study: SS harvested from 4-day-old liquid culture and AS from a one-week-old SSF. All samples were fixed for one hour in 2.5% of glutaraldehyde 0.1 M phosphate buffered saline (PBS) at pH 7.4. After fixation, the suspensions were centrifuged and washed thrice for 5 min in distilled water to remove chemicals, then incubated for 1 hour at room temperature in a 1%-osmium tetroxide solution (1%-OsO4), washed thrice in distilled water for 5 min and finally dehydrated through an ethanol-water series (baths of 5 min each) until absolute ethanol (3x20 min) and finally propylene oxide (3×20 min). Sporidia were soaked for 3 hours in propylene oxide epoxy resin mixture (1:1 v/v), then embedded overnight in a hard mixture of epoxy resin, AGAR-Low-Viscosity Resin Kit (AGAR, R1078, Agarscientific, United Kingdom). For polymerization step, the samples were kept for 4 days at 60°C.

Ultra-thin sections (80–100 nm) were made using a 3mm-diamond knife (Diatom) on a Reichert-Jung Ultracut E ultramicrotom, put copper grids and contrasted with uranyl acetate (1% in ethanol 50%) and lead citrate according to the routine procedure before examination in a Jeol JEM 100-SX transmission electron microscope operating at 80 kV.

### Germination tests

Coverslips (15x15 mm) were first sterilized, coated on one side with YMPDA with addition of chloramphenicol (10 μg/l) and cooled on a glass slide which was placed in a Petri dish lined with moist filter paper (to avoid air-drying).

Ten μl from AS suspension (10^7^ sporidia/ml) was deposited onto the coated coverslip and spread lightly with a glass rod. Coated coverslips were dropped in the moist Petri dish which was sealed with a strip. The same step was repeated for SS suspension. The coverslip cultures were incubated at 25°C. The germinating cultures were observed under the light microscope every hour for 7 h. Sets of 50 SS and AS were used to calculate means and standard deviations in triplicate (three sets of 50 sporidia). In each set, the germinated AS and SS were determined.

### Melanin extraction

According to the method of Gadd [32], melanin was extracted from 5 g of each AS and SS samples. Those samples were washed in distilled water and then in 1 M NaOH, followed by a centrifugation at 4000 g at room temperature. The melanin molecules were extracted by boiling for 20 min in 5 ml of 1 M NaOH, followed by autoclaving for 20 min at 120°C.

After removal of cell debris by centrifugation at 12000 g for 5 min, the supernatant was treated with the organic solvents chloroform, ethyl acetate, and ethanol (1 ml each) to remove carbohydrates and lipids.

The pigments were precipitated by acidifying to pH 2 with HCl (7M) and centrifuged for 20 minutes at 12000 g. The pellet was washed with distilled water and freeze-dried.

### UV-visible spectrophotometer and flow cytometry

Purified pigments were dissolved in 1 ml of 1 M NaOH. The UV-visible absorption spectra were recorded in the wavelength range from 200–600 nm using UV-Vis spectrophotometer (*Genesys 10S UV*-*Vis*, Thermo Scientific) and compared with those of synthetic DOPA (3,4-dihydroxyphénylalanine) melanin (Sigma, M8631, Germany).

The fluorescence intensity of melanin was also quantified by flow cytometry on the FL1 channel of a C6 Accury Flow Cytometer (BD Biosciences, NJ, USA). The sample of AS were diluted with phosphate buffered saline (PBS, pH = 7) to adjust the cell density between 500 and 2500 events/μL during flow cytometry analysis.

The sporidia were gated on the forward scatter/side scatter (FSC/SSC) plots. Forty thousand cells per sample were analyzed and the mean fluorescence intensity was recorded. C-Flow software was used for Flow cytometry data analysis.

### Relative fluorescence intensity

Relative fluorescence intensity (RFU) was measured using SpectraMax equipment (Spectramax M2e, Molecular devices, USA) in a 96 well, UV-star, transparent microplate with a working volume of 25 to 340 μl (Greiner Bio-One) at an excitation wavelength range of 200–600 nm and 550 nm of emission wavelength.

### Statistical analysis

Statistical analysis was conducted using one-way analysis of variance (ANOVA) using R version 3.5.0 software. P-values were considered significant if lower than 0.05.

## Results

### Shape and size of aerial and submerged sporidia

**Fig 1:**
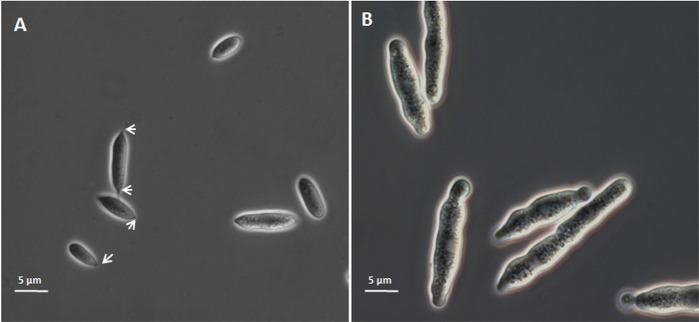
*Pseudozyma flocculosa* sporidia in phase contrast microscopy. A: aerial sporidia (AS) obtained by solid-state fermentation, exhibiting one or two apiculate hilum (asterisks). B: submerged sporidia (SS) obtained by liquid-state fermentation, regularly showing protrusion bulging and inclusions, Arrows point to the apiculate hilum of the AS.

Light microscopy (LM) images already revealed significant differences in shape and size between AS and SS (Fig 1). SS appeared much longer (15,4 ± 1,6 μm) (*P*<0,001) and wider (2,8 ± 0.8 μm) (*P*<0,05) than AS (8,2 ± 3 μm long and 2,3 ± 0.6 μm width). Regarding to shape, SS showed rounded edges and endings that regularly formed small protrusion bulgings. The end of AS showed apiculate hilum (one or two) that are interpreted as sporidia scars (Fig 1A), *i*.*e*. one for terminal sporidia and two for in-filament sporidia. Furthermore, there was no inclusion visible in the cytoplasm of AS.

**Fig 2:**
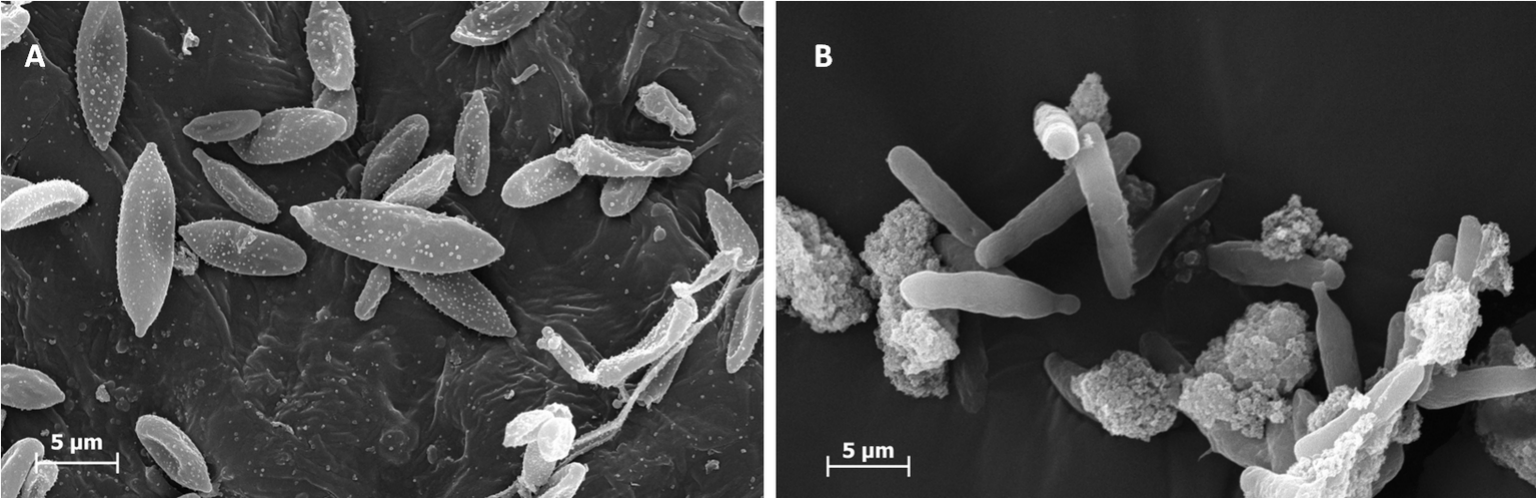
SEM images of *Pseudozyma flocculosa* sporidia. A: aerial sporidia produced by solid-state fermentation; B: submerged sporidia from the liquid-state fermentation.

Scanning electron microscopy (SEM) imaging confirmed the shape of sporidia as seen in LM. The images showed that the AS are fusiform (spindle-shaped) with hilum at one or two of the end. Most sporidia had concave deformations, interpreted as dehydration artefacts (Fig 2A).

**Fig 3:**
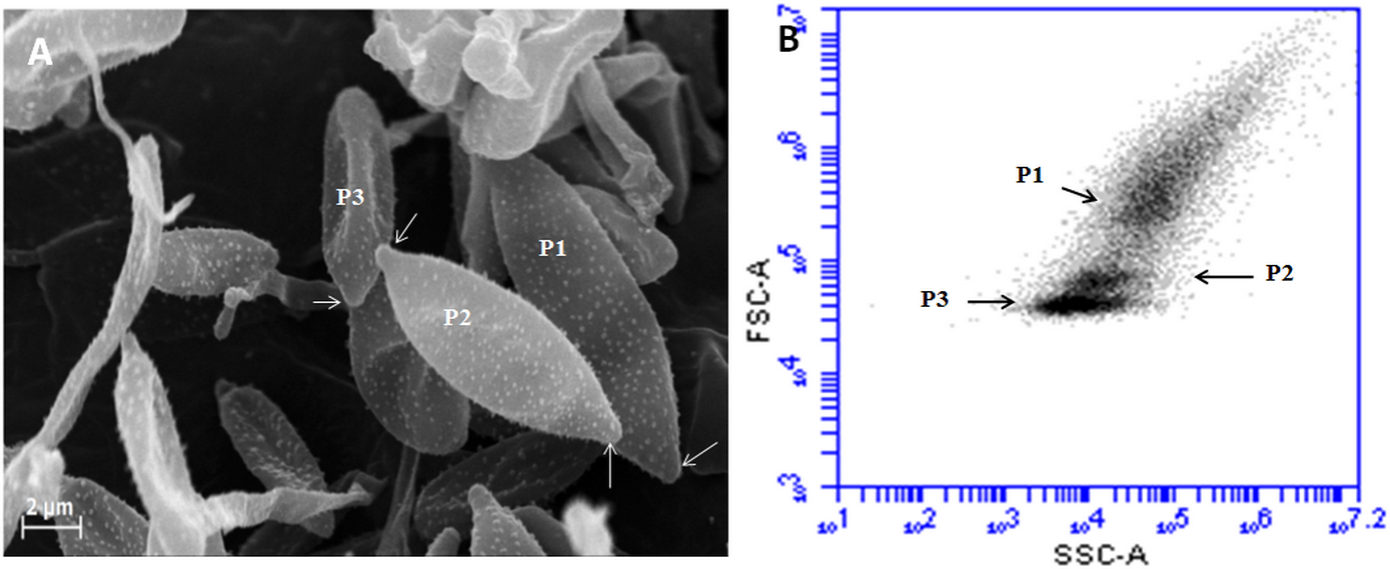
SEM views of the three different populations of aerial sporidia. A: Large sporidia (P1), medium-sized sporidia (P2) with two scars (arrows) and small sporidia (P3) with one scar only. B: Flow cytometry analyses corresponding to the AS sample, revealed different morphotypic sub-populations (results were displayed on Forward Scatter Air/Forward Scatter Height (FSC-A/FSC-H) dot-plot on the basis of 40,000 events).

In addition, SEM-imaging and flow cytometry showed that AS are subdivided into three populations, characterized by shape and size. The first population (P1) consists of large sporidia about 12,3 ± 1,5 μm long and 3,5 ± 0,54 μm broad, with 2 scars (Fig 3P1). The sporidia of the second population (P2) are medium-sized, 8,9 ± 0,7 μm long and 2,5 ± 0,38 μm broad, with 2 scars (Fig 3P2). The third population (P3) contains the smallest sporidia, 5 ± 1 μm long and 1,65 ± 0,5 μm broad, with a single scar only and a round-shaped end (Fig 3P3). The difference between the three populations is clearly significant according to the ANOVA test (*P* <0.001).

The SS always were longer than aerial sporidia, 16 ± 2,5 μm (*P* <0.001). But the difference in thickness between SS (2,5 ± 0,4 μm) and AS (P2) was not significant (*P*>0,05). SS were cylindrical with rounded edges and endings resembling buds more than sporidial scars.

### Life cycle of *Pseudozyma flocculosa*

**Fig 4:**
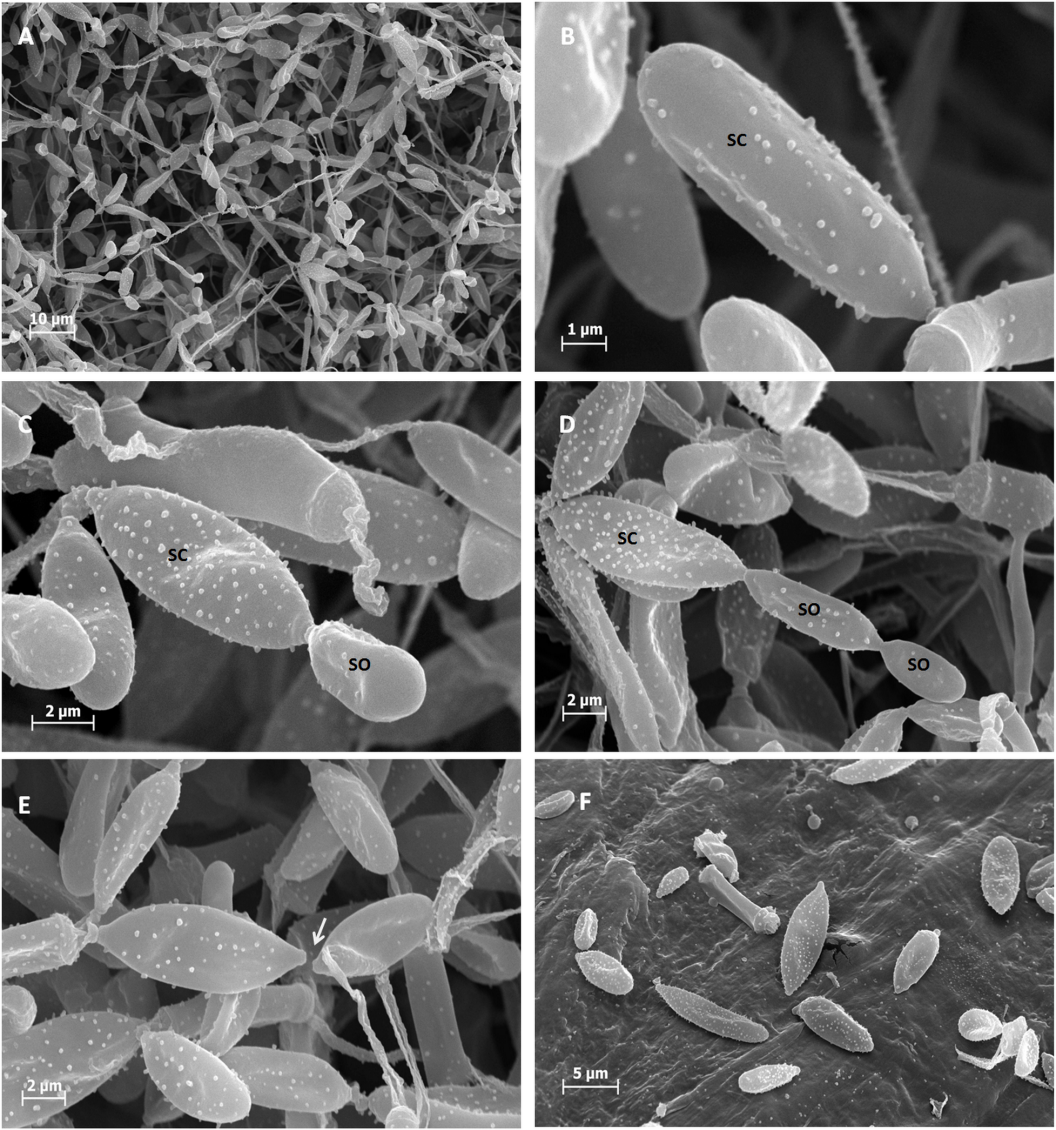
Sporogenesis of *Pseudozyma flocculosa*. A: General view of Aerial sporidia, B: sporidiogenous cells (sc) on a mycelial branch (m), C: sporidiogenous cells at different stages of development, D: sporidiogenous cells with two sporidia (so) forming a chain of three sporidia, E: Healing of sporidia with a sporidial scar (white arrow), F: pollination of sporidia.

SEM observations of SSF samples clearly show that this fungus sporulates by forming sporidiogenous cells (mother cell) with buds on the various branches of the mycelium. These large sporidiogenous cells (Fig 4A) give rise to one or two cell types (Fig 4B and 4C) of smaller size, and form a chain of two to three sporidia. After their maturation, these sporidia eventually break away and keep the hilum as a scar from the sporogenesis. This type of sporogenesis is named “acroblastosporae” [29].

### Internal and wall ultrastructure of sporidia

Differences in the external surface, wall thickness and structure and cytoplasmic content of the sporidia were also apparent and related to the growing conditions of sporidia.

**Fig 5:**
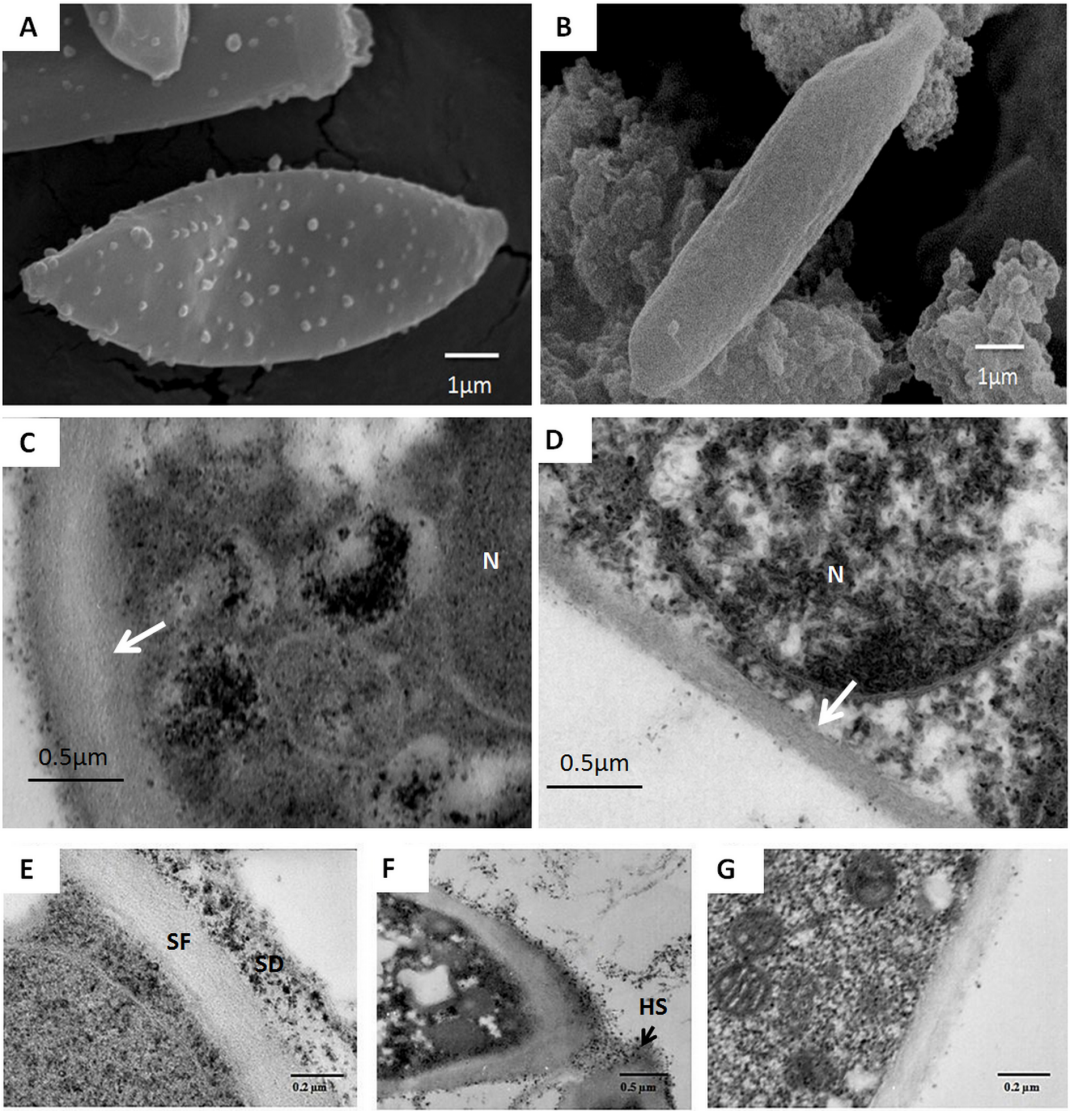
SEM and TEM images of sporidia wall surface. A: aerial sporidia (AS) wall with ornament granules. B: submerged sporidia (SS) with a smooth wall surface. C: thick walls of AS, D: thin wall (white arrows) of SS with clear nucleus (N). E: thick walls of AS with an inner fibrous electron-lucent layer (SF) and an outer granular electron-dense layer (SD) showing a high affinity for the contrasting agents, F: hilum of sporogenesis (HS) of AS and G: Thin electron-lucent wall of SS.

The AS walls exhibited a verrucous surface dotted with small ornament granules (Fig 5A). In TEM (Fig 5C), the AS walls appeared relatively thick (approximately 400 nm), due to the presence of an important outer granular electron-dense layer (Fig 5E and 5F) overlaying the inner fibrous layer. The granules in this layer are due to a strong reaction with contrasting agents, especially OsO_4_ that is known to stain unsaturated lipids (as those of biological membranes) and other reducing molecules [33].

In these sporidia, the cytoplasm was strongly electron-dense with poorly distinguishable organelles except some electron-lucent stored materials (Fig 5C and 5D). The nucleus appeared discrete, mostly including condensed chromatin and a discrete nucleus.

In contrast, the wall of SS showed completely smooth surface without any relief (Fig 5B), and appeared much thinner than that of AS. The cell wall includes only an inner electron-lucent layer not exceeding 100 nm and a very thin outer dense layer (few nm) without/or with very few dense granules (Fig 5D and 5G). The cytoplasm of SS appeared classically electron-lucent with a granular texture and easily recognizable organelles.

### Auto-fluorescence

**Fig 6:**
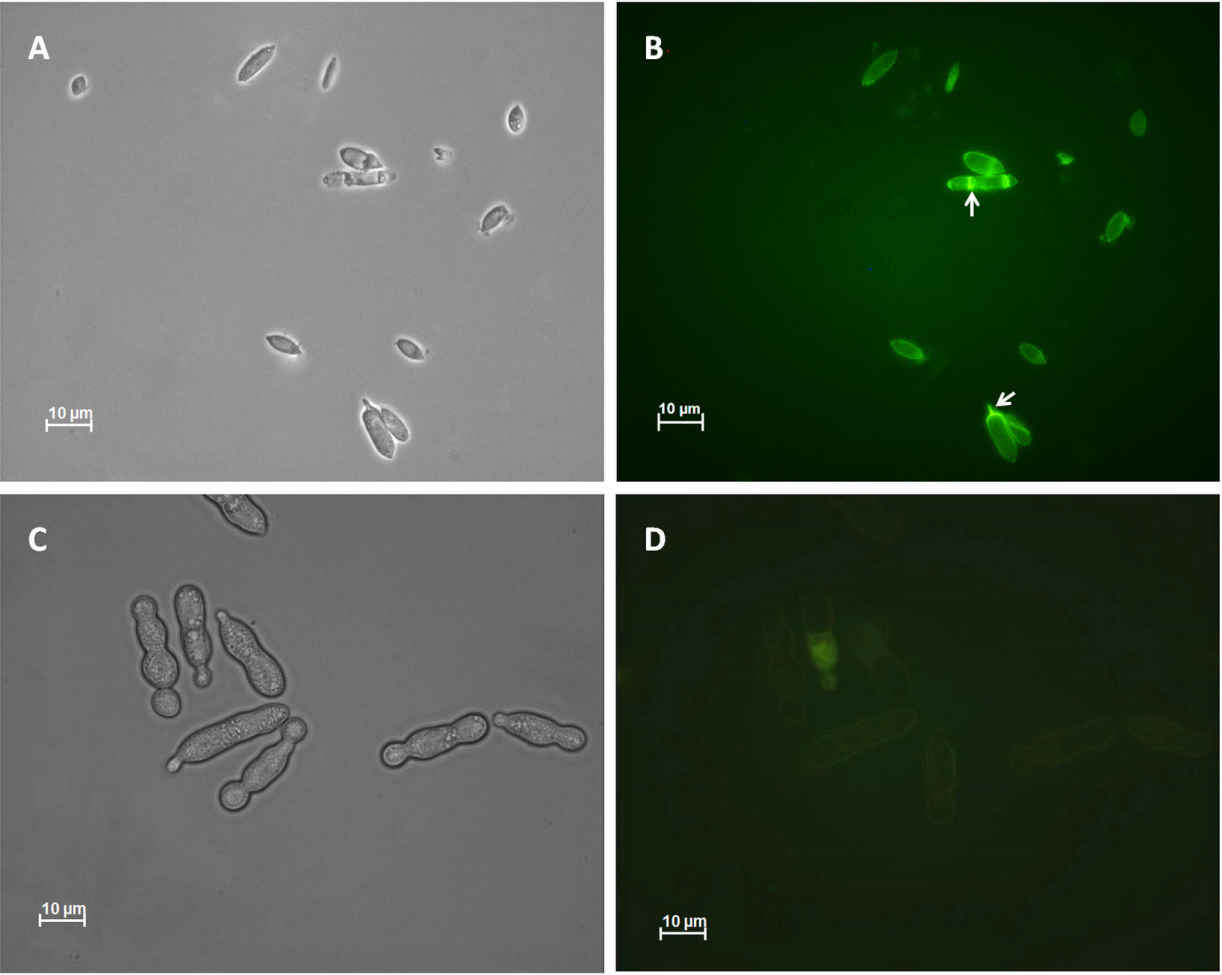
Auto-fluorescence of cell wall of sporidia. A and B: AS recovered from 12-day-old cultures in solid-state fermentation. C and D: SS grown in liquid-state fermentation. Arrows show the sporidia hila.

The comparative observation of two types of sporidia by fluorescence microscopy showed a strong auto-fluorescence emission at the periphery of AS and locally inside the cytoplasm. It corresponds to cytoplasmic inclusions and very likely to the sporidia walls (Fig 6A and 6B) and was particularly intense at the sporidial hila. In contrast, SS emitted a very weak and diffuse fluorescence, also at their periphery (Fig 6C and 6D) and rarely inside the cells.

### Melanin

Most hyphal and conidial or sporidial walls of melanized fungi appear to have two distinct layers: an inner layer that is electron translucent and an outer layer that contains and may be covered by electron-dense granules [34]. Ghamrawi *et al*. (2014) showed that the outer layer of *Pseudallescheria boydii* conidia is composed of melanin molecule [35]. This indicates that the outer layer that is missing in SS could be melanin.

**Fig 7:**
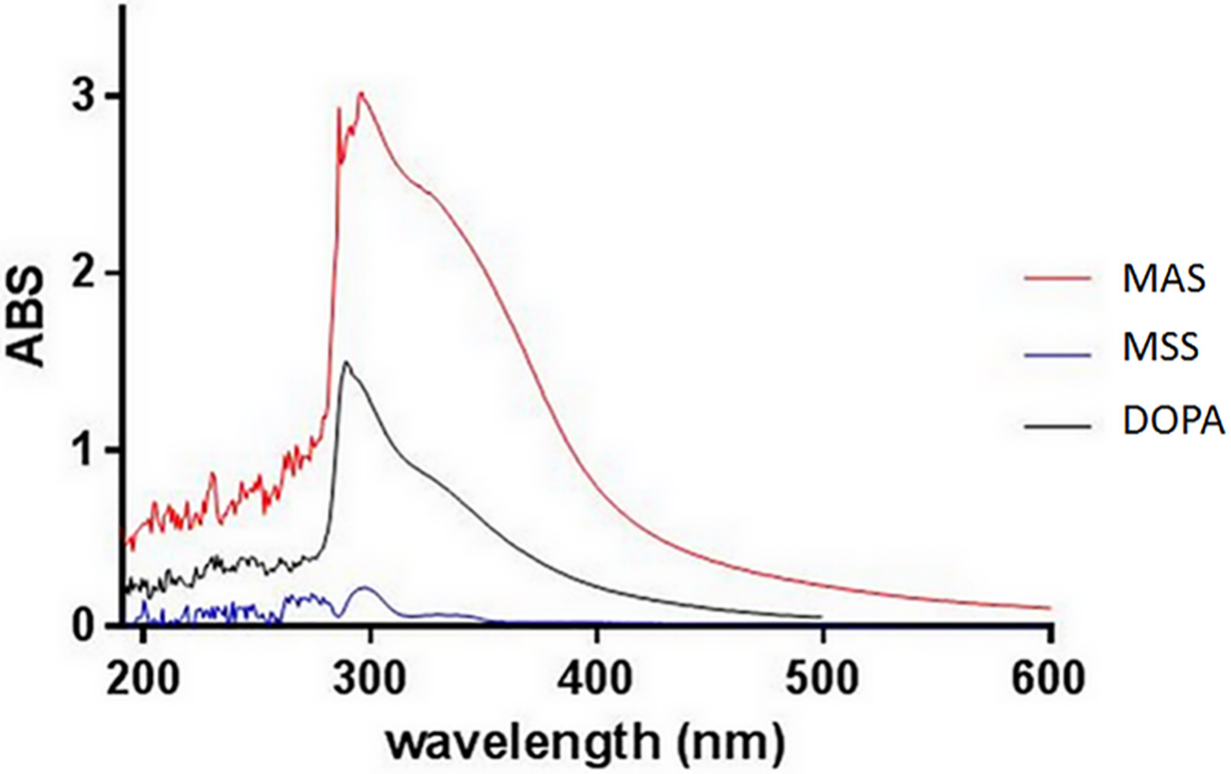
UV-visible spectrophotometry of melanin extracts. MAS: Melanin extracts from aerial sporidia, MSS: Melanin extracts from submerged sporidia, DOPA: synthetic melanin. ABS: absorbance.

To confirm this hypothesis UV absorption spectra and fluorescence spectra of melanins extracted from the two types of sporidia (AS and SS) of *P*. *flocculosa* were compared to a synthetic DOPA melanin. UV absorption spectra revealed high melanin content in AS compared to SS with an absorption maximum at 296 nm of 3 ± 0,01 AU (absorbance unit) for AS and 0,2± 0,003AU for SS (*P*<0,001) (Fig 7).

**Fig 8:**
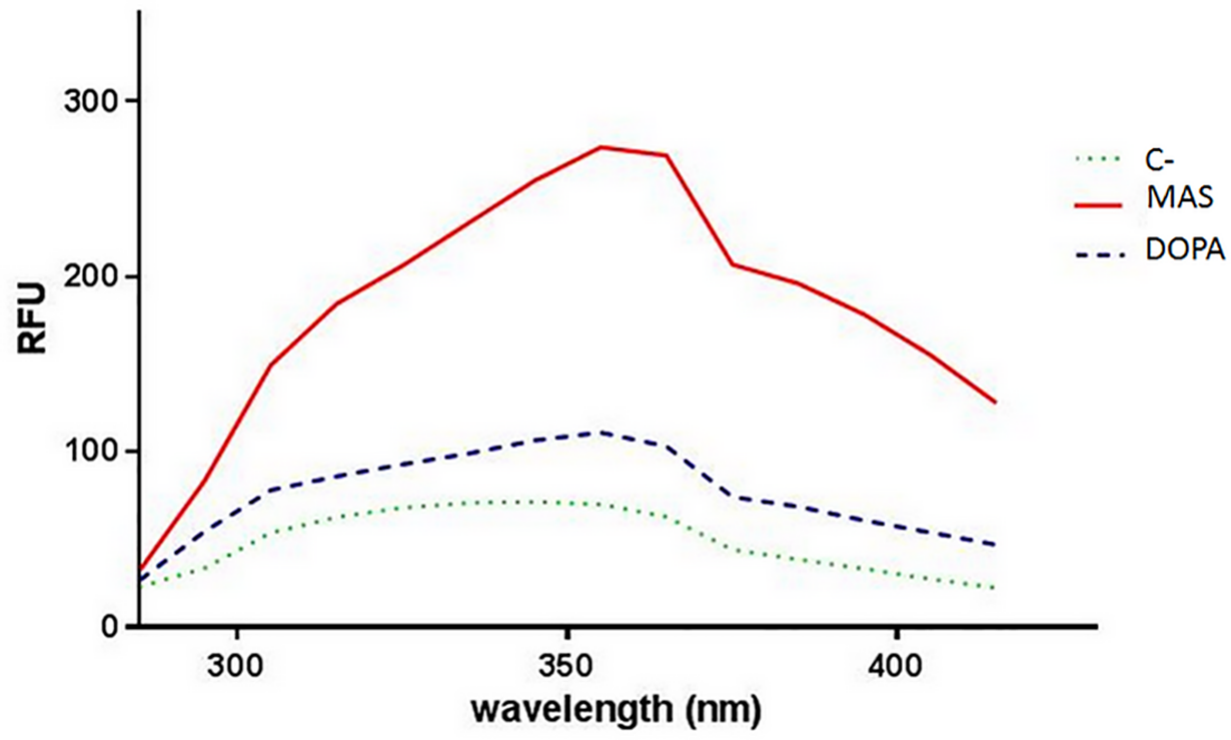
Fluorescence spectra obtained by SpectraMax M5 in the 300–440 nm wavelength range. MAS: melanin extracts from aerial sporidia, DOPA: sample containing a synthetic melanin (DOPA melanin) (0.5mg/ml), C-: reference sample without melanin. RFU: Relative fluorescence unit.

The relative fluorescence spectrum of AS melanin obtained in the 300–440 nm wavelength range with a SpetraMax M2 showed a very similar spectral shape to DOPA melanin. The fluorescent emission of melanin from AS was much stronger than that of DOPA, reaching 269,2 ± 33 RFU compared to 103 ± 16 RFU for synthetic DOPA melanin (*P*< 0,001) (Fig 8). This difference is probably due to differences in the composition of the melanin. Because of the close genetic link between *Ustilago maydis* and *P*. *flocculosa* [36], we could positively estimate that the melanin type of the latter belongs to the catechol melanin group[34].

### Cytoplasm and nucleus difference

**Fig 9:**
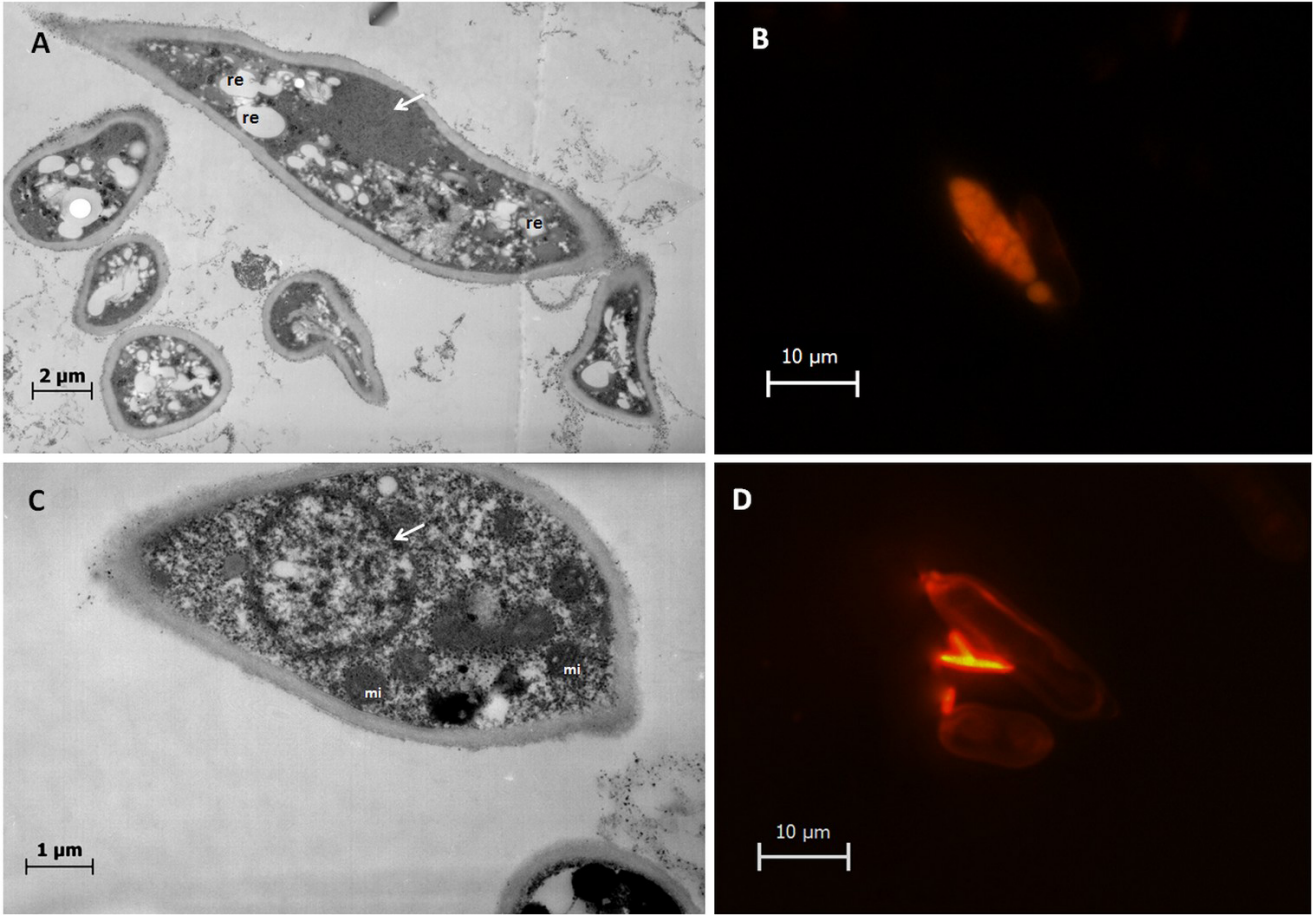
Differences in the cytoplasm of aerial sporidia (AS) and submerged sporidia (SS). The sporidia were colored by Nile-red fluorescent dye marking the intracellular cells lipids. A and B: AS have a dense compact nucleus (white arrows) with large lipid stocks. C and D: SS are rich in mitochondria organelle and have a clear nucleus (white arrows).

TEM observation clearly shows differences in the cytoplasm and nucleus between AS and SS. AS were fully charged with inclusions which were not limited by membranes and dispersed throughout of the cytoplasm. The staining with Nile red fluorescent dye showed that the inclusions were lipid stocks. Moreover, AS had a dense and compact nucleus (Fig 9A and 9B). On the other hand, SS lacked these inclusions and had a clear nucleus and a cytoplasm rich in mitochondria (Fig 9C and 9D).

### Germination test

To understand if the great morphological difference between the two types of sporidia has an effect on the germination time of sporidia. A comparative test of germination was achieved between the AS and SS.

**Fig 10:**
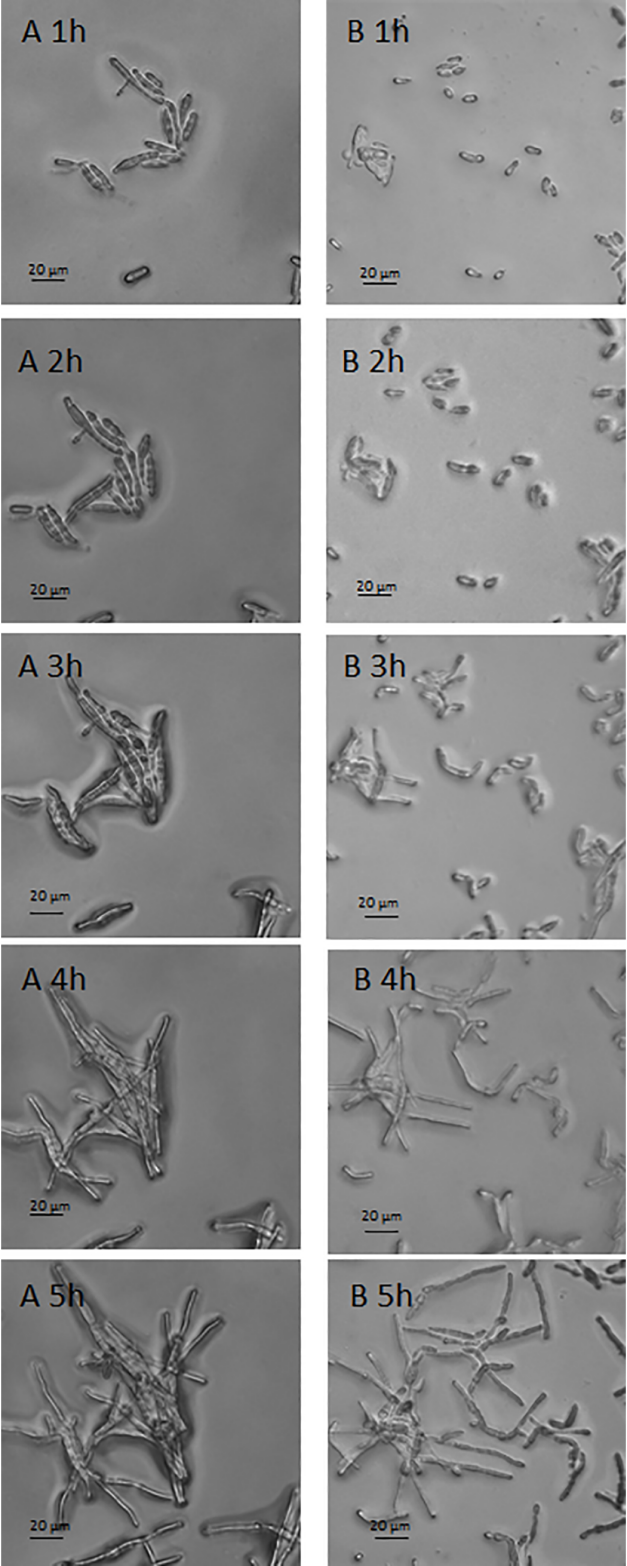
Germination test. A series: germination of submerged sporidia. B series: Germination of aerial sporidia. Numbers correspond to test time, expressed in hours (h).

In the first two hours, no evolution was observed in AS, while a majority of the SS (93 ± 2,3%) of *P*. *flocculosa* has already grown several micrometers in length and began to germinate (Fig 10A1 and 10A2). After three hours of onset of test, the majority of AS (91,1 ± 4,2%) began to grow, whereas SS have all germinated and transformed into non-branching filaments (Fig 10A3 and 10B3). After 5 hours, the SS gave mycelium and AS began to transform into non-branching filaments (Fig 10A4, 10B4 and 10A5, 10B5)

## Discussion

The knowledge of the physiological form and structural changes in cytoplasmic organelles and cell walls of *P*. *flocculosa* sporidia from liquid- and solid-state fermentation is crucial to understand the main cause of resistance and susceptibility of these sporidia against hostile environmental conditions.

Most studies previously performed on *P*. *flocculosa* were devoted to its antagonistic effects against the disease of powdery mildew. But the origin of the low resistance of sporidia from a submerged fermentation remains unknown and problematic. Up to now, all production of sporidia or conidia have been made with this technique because of its economic advantages, short production time, and lower risks of contamination.

In this study many of differences have been highlighted between AS and SS. The microscopic results show that there is a difference in size and shape between the AS and SS of *P*. *flocculosa*. This difference was also confirmed by Yu-Hui on *Pseudozyma antartica* [37]. First the sporogenesis cycle results in heterogeneity in size and shape of the AS. When forming, the three-sporidia chains as observed in SEM begins with a large sporidia corresponding to a sporidiogenous cell which will itself produce another sporidia by budding and so on up to the formation of the chain. This type of sporogenesis is named acroblastosporea [29].

Moreover, significant differences in the cytoplasm and cell wall structure could really explain the high resistance and slow germination of AS and, by contrast, the high susceptibility and rapid germination of SS as previously shown for other fungi [25,35,38].

The auto-fluorescence is only observed from the walls of the AS. This characteristic indicates that there is a difference in the composition of the wall between the sporidia from the two propagation techniques. This was confirmed by TEM, by the presence of an electron-dense outer layer in the AS. According to the literature a mature conidium or sporidium is ornamented and thick-walled [29]. Naturally conidia or sporidia are probably better protected by the development of a thick outer layer enriched in melanin. Indeed melanized-wall of AS consists of two distinct layers: an inner electron-lucent layer and an outer layer charged with electron-dense granules that can correspond to melanin pigments [34].

The melanin extraction and spectroscopy data from AS and SS demonstrate that melanin is only present in substantial amounts in AS and fluoresces in the 250–400 nm range, while melanin is almost absent in SS. This provides strong evidence that the presence of melanin is related to the auto-fluorescence and to the development of the thick granular layer specific of the AS walls. Moreover, due to the reducing properties of melanin compounds, melanin likely react with the OsO_4_-fixative agent and then appear as black-stained grains in the outer layer [33]. Melanin and auto-fluorescence are almost absent in the SS walls that only possess a smooth wall with a reduced non-granular outer layer. The relationship between fluorescence and melanin has already been shown in human melanin where there was an induction of auto-fluorescence under UV light from 340 to 400 nm [39,40].

Melanization in fungi is likely to provide a survival advantage in the aerial environment. The outer layer formed by the melanin protects microorganisms against a broad range of toxic insults [41]. Consequently, this molecule has been called fungal armor [42]. For instance, the outer melanin layer in *Cryptococcus neoformans*, reduces its susceptibility to enzymatic degradation and toxicity from heavy metals. This layer also protects *Pseudallescheria boydii* against host immune defenses by masking the mannose-containing glycoconjugates that are involved in immune recognition [22,35,43].

Melanin is a natural pigment produced by the oxidation of the tyrosine amino-acid (DOPA melanin) or other precursors such as γ-glutaminyl-4-hydroxybenzene (GDHB Melanin), Catechol (catechol melanin) and 1,8-dihydroxy-naphthalene (DHN melanin), followed by polymerization. Melanin is an effective absorber of UV light. The pigment is able to dissipate over 99.9% of absorbed UV radiation [44]. Because of this property, melanin is thought to protect conidia or sporidia cells from UVB radiation damages, protecting their genetic material [21,41]. The absence of melanin in outer layer of SS could be the main cause of its low resistance against unfavorable factor. The absence of this molecule may be making SS unprotected against the UV radiation. This also increases the susceptibility of SS to environmental conditions.

The presence of a thin non-melanized wall in SS with but a very-reduced outer layer indicates that they may not have achieved complete structural maturation of walls as the AS do. A recent study performed on *P*. *boydii* presented changes in biochemical composition of the conidial wall with the age of culture, highlighting the process of conidial maturation [35].

The presence of mitochondria in the cytoplasm of *P*. *flocculosa* SS with an absence of metabolic reserves indicates that the compartmentalization process is not yet complete. This explains the rapid germination in SS and confirms that SS do not reach their dormant state. An incomplete maturation in the liquid-state fermentation could be the cause of its susceptibility to adverse conditions.

In conclusion, AS and SS of *P*. *flocculosa* are dissimilar in a number of aspects, spore size and spore germination. The complete dormancy in AS with densification of cytoplasm and nucleus, reduced metabolism, thick protective cell walls, and enriched in melanin may be responsible for their stability. The fact that SS do not reach this stage of dormancy could be the main cause of their susceptibility to adverse conditions.

A study of the biochemical changes in the sporidial wall in *P*. *flocculosa* and a proteomic comparison between two types of sporidia during sporogenesis could help a better understanding of the main cause of incomplete maturation in SS of *P*. *flocculosa* and open the door to improving the quality of this type of sporidia for future use as biocontrol tool. Moreover, the impact of observed differences between SS and AS on biocontrol of powdery mildew should be investigated in a future study.
